# Supplementary material for: Structures of Human Antibodies Bound to SARS-CoV-2 Spike Reveal Common Epitopes and Recurrent Features of Antibodies
Source: Cell. 2020 Aug 20;182(4):828–842.e16. doi: 10.1016/j.cell.2020.06.025 (PMC7311918; doi:10.1016/j.cell.2020.06.025)
Supplement: Document S1. Tables S1–S3 [file mmc1.pdf]

Cell, Volume *182*

Supplemental Information

Structures of Human Antibodies Bound to SARS-CoV-2 Spike Reveal Common Epitopes and Recurrent Features of Antibodies

Christopher O. Barnes, Anthony P. West Jr., Kathryn E. Huey-Tubman, Magnus A.G. Hoffmann, Naima G. Sharaf, Pauline R. Hoffman, Nicholas Koranda, Harry B. Gristick, Christian Gaebler, Frauke Muecksch, Julio C. Cetrulo Lorenzi, Shlomo Finkin, Thomas Hägglöf, Arlene Hurley, Katrina G. Millard, Yiska Weisblum, Fabian Schmidt, Theodora Hatziioannou, Paul D. Bieniasz, Marina Caskey, Davide F. Robbiani, Michel C. Nussenzweig, and Pamela J. Bjorkman

Supplemental Tables

Table S1. Crystallographic data collection and refinement statistics for C105 Fab structure (related to Figure 5).

	**C105 Fab** *(12-1, SSRL)*
**PDB ID**	**6XCA**
**Data collection**[a]	
Space group	I222
Unit cell (Å)	67.4, 120.1, 123.3
α, β, γ (°)	90, 90, 90
Wavelength (Å)	1.0
Resolution (Å)	38.9-1.80 (1.84-1.80)
Unique Reflections	46,713 (2752)
Completeness (%)	100 (99.8)
Redundancy	6.8 (6.5)
$CC_{1/2}$ (%)	98.8 (54.1)
<I/σI>	5.7 (1.2)
Mosaicity (˚)	0.19
R_{merge} (%)	18.1 (157)
R_{pim} (%)	7.9 (70.5)
Wilson *B* -factor	16.8
**Refinement and Validation**	
Resolution (Å)	38.9 - 1.80
Number of atoms	
Protein	3,132
Ligand	10
Waters	477
R_{work}/R_{free} (%)	18.7/21.6
R.m.s. deviations	
Bond lengths (Å)	0.006
Bond angles (˚)	0.853
MolProbity score	1.29
Clashscore (all atom)	4.2
Poor rotamers (%)	0
Ramachandran plot	
Favored (%)	97.6
Allowed (%)	2.4
Disallowed (%)	0
Average *B* -factor (Å)	27.1

[a]Numbers in parentheses correspond to the highest resolution shell

Table S2. Cryo-EM data collection and refinement statistics for C105-S complex structure (related to Figure 5).

	C105 SARS-CoV-2 S 2P (state 1)	C105 SARS-CoV-2 S 2P (state 2)
**PDB**	**6XCM**	**6XCN**
**EMD**	**22127**	**22128**
Microscope	Titan Krios	Titan Krios
Camera	Gatan K3 Summit	Gatan K3 Summit
Magnification	105,000x	105,000x
Voltage (kV)	300	300
Recording mode	counting	counting
Dose rate (e^-/pixel/s)	22.1	22.1
Electron dose (e^-/Å^2)	60	60
Defocus range (μm)	1.0 - 2.5	1.0 - 2.5
Pixel size (Å)	0.418 (super resolution); 0.836 (binned)	0.418 (super resolution); 0.836 (binned)
Micrographs collected	5,940	5,940
Micrographs used	5,336	5,336
Total extracted particles	71,289	71,289
Refined particles	57,710	14,119
Symmetry imposed	C1	C3
Nominal Resolution (Å)		
FSC 0.5 (unmasked/masked)	3.90/3.60	4.00/3.60
FSC 0.143 (unmasked.masked)	3.40/3.20	3.50/3.40
Map sharpening *B* -factor		
**Refinement and Validation**		
Number of atoms		
Protein	25,973	27,798
Ligand	711	873
MapCC (global/local)	0.86/0.84	0.87/0.85
R.m.s. deviations		
Bond lengths (Å)	0.008	0.011
Bond angles (˚)	0.812	0.893
MolProbity score	2.17	2.26
Clashscore (all atom)	13.6	14.8
Poor rotamers (%)	0.04	1.1
Ramachandran plot		
Favored (%)	90.9	89.9
Allowed (%)	9	10.1
Disallowed (%)	0.1	0

Table S3. S protein mutations found in different SARS-CoV-2 isolates (related to Figure 6).

Mutation	Count	Frequency(%)	Location
D614G	9688	63.2	S1 domain D
P1263L	115	0.7	S2 cytoplasmic tail
L5F	91	0.6	signal sequence
D936Y	88	0.6	S2 HR1
L54F	58	0.4	S1 domain A
G1124V	56	0.4	S2
N439K	38	0.2	S1 domain B (RBD)
H49Y	35	0.2	S1 domain A
L18F	31	0.2	S1 domain A
L8V	30	0.2	signal sequence
A831V	29	0.2	S2
D839Y	28	0.2	S2
V483A	28	0.2	S1 domain B (RBD)
Q675H	24	0.2	S1 domain D
S50L	24	0.2	S1 domain A
S943P	22	0.1	S2 HR1
A1078S	21	0.1	S2
R21I	19	0.1	S1 domain A
V367F	18	0.1	S1 domain B (RBD)
T29I	18	0.1	S1 domain A

List of SARS-CoV-2 spike mutations with a frequency ≥ 0.1% in a set of 15335 isolates downloaded from the Global Initiative for Sharing All Influenza Data (GISAID) SARS-CoV-2 sequence database on 5/3/20 (Elbe and Buckland-Merrett, 2017; Shu and McCauley, 2017). The genomes were processed with the nextstrain augur pipeline (https://github.com/nextstrain/augur) (Hadfield et al., 2018), using MAFFT v7.464 (Katoh and Standley, 2013) for sequence alignment and FastTree (Price et al., 2010) to generate a phylogenetic tree. The resulting data were then analyzed with a custom Swift program.
